# Gelatin from Saithe (*Pollachius virens)* Skin: Biochemical Characterization and Oxidative Stability in O/W Emulsions

**DOI:** 10.3390/md20120739

**Published:** 2022-11-25

**Authors:** Betül Yesiltas, Chloé Robert, Heidi Olander Petersen, Flemming Jessen, Fatemeh Ajalloueian, Mohammad Amin Mohammadifar, Charlotte Jacobsen, Jens J. Sloth, Greta Jakobsen, Federico Casanova

**Affiliations:** 1 Research Group for Bioactives—Analysis and Application, National Food Institute, Technical University of Denmark, Kemitorvet, 2800 Kongens Lyngby, Denmark; 2 Research Group for Food Production Engineering, National Food Institute, Technical University of Denmark, Søltofts Plads, 2800 Kongens Lyngby, Denmark; 3 Agrocampus Ouest, UMR 1253, F-35042 Rennes, France; 4 Center for Intelligent Drug Delivery and Sensing Using Microcontainers and Nanomechanics (IDUN), Department of Health Technology, Technical University of Denmark, 2800 Kongens Lyngby, Denmark; 5 Research Group for Analytical Food Chemistry, Technical University of Denmark, Kemitorvet, 2800 Kongens Lyngby, Denmark; 6 Danish Fish Protein, Adelvej 11, Hoejmark, 6940 Lem, Denmark

**Keywords:** fish skin gelatin, physical and oxidative stability, oil-in-water emulsions

## Abstract

This study performed the extraction of gelatin from saithe (*Pollachius virens*) skin and compared it to commercial marine gelatin. As a first stage, we investigated the physicochemical and biochemical properties of the gelatin. SDS-PAGE analysis revealed the presence of α-chains, β-chains, and other high-molecular-weight aggregates. DSC thermograms showed typical gelatin behavior, while the FTIR spectra were mainly situated in the amide band region (amide A, amide B, amide I, amide II, and amide III). In the second stage, we produced O/W emulsions and analyzed their physical and oxidative stability over 9 days. Oil droplets stabilized with the gelatins obtained from saithe fish skin had a size of ~500 nm and a ζ-potential ~+25 mV, which is comparable to oil droplets stabilized with commercial gelatin products. Moreover, the oxidative stability of the emulsions stabilized with gelatin from saithe fish skin showed promising results in terms of preventing the formation of some volatile compounds towards the end of the storage period compared to when using the commercial gelatins. This study indicates the potential application of fish skin gelatin in the fields of food and cosmetics, as well as suggesting that further investigations of their techno-functional properties.

## 1. Introduction

An emulsion is a colloidal system in which one immiscible liquid is dispersed in another liquid, forming micrometric or nanometric droplets that are thermodynamically unstable [1]. Various physicochemical mechanisms, such as coalescence, separation by gravitational force, flocculation, and Ostwald maturation, can occur, ultimately leading to the separation of the water and oil phases. In the food industry, proteins are widely employed to (i) stabilize food emulsions during storage via steric and/or electrostatic repulsions and (ii) inhibit lipid oxidation, which is a serious threat to quality, as it leads to the degradation of the nutritional quality of the product and generates undesirable flavors [2,3]. In addition, the use of natural antioxidants is of great interest to food manufacturers and researchers in the current context of high demand among consumers for sustainable, health-promoting, and clean-label food [4].

Nowadays, proteins from dairy or egg sources are typically employed as emulsifiers and stabilizers; however, in the actual context of protein transition, there is increasing interest in finding more sustainable sources. For instance, gelatin from marine sources has become a new alternative in the food industry [4,5,6]. Fish gelatin is (i) a clean-label product that is not encumbered by religious restrictions and (ii) an ingredient that is obtained through the valorization of byproducts of marine sources [5,6]. In the North Atlantic Sea, saithe is an economically important fish species, and has an annual catch of around 300,000 tones. Normally, saithe are used for fillet production, consequently generating a large amount of byproducts.

Previous studies have validated the fact that gelatin from saithe skin can be used as a stabilizer in emulsion systems, and the authors have previously demonstrated that different extraction processes can affect their physical stability [7]. However, no investigations have been conducted on the oxidative stability of O/W emulsions stabilized with gelatin from fish skin obtained using different extraction protocols. To address this, the following work is divided into two main sections. In the first part, two different saithe skin gelatin products are extracted and investigated in terms of their physico-chemical, thermal and structural properties, in comparison to two commercial gelatin products. We determined the moisture, ash, protein content, and mineral composition and used SDS-PAGE to determine the protein profile. Fourier transform infrared (FTIR) spectroscopy was employed to study the changes in functional groups and secondary structures of gelatin, whereas differential scanning calorimetry (DSC) was used to explore their thermal properties. In the second part, we produced O/W emulsions, and we analyzed their physical and oxidative stability during storage for nine days. The physical stability of the emulsions was explored using the Turbiscan tower, and the droplet size and surface charge of the droplets were analyzed using laser diffraction and dynamic light scattering (DLS) techniques. Finally, the oxidative stability of the emulsions was evaluated based on the formation of hydroperoxides and secondary volatile oxidation products, and consumption of tocopherols.

## 2. Results

### 2.1. Physico-Chemical Properties and Mineral Composition

Table 1 presents the physico-chemical properties, i.e., moisture, ash, protein content, gel strength and mineral composition of the fish gelatin powders. Samples G2, G3 and G4 present moisture content between 5.08 and 7.18% whereas sample G1 shows a value of ~12%. The ash results revealed the presence of two different groups, with samples G1 and G2 having values lower than 0.21% and samples G3 and G4 having values higher than 2%. As reported by Alfaro et al. [8], the maximum ash content recommended for human consumption is 2.6%. No significant difference was observed in terms of protein content, and the values were ca. 95%. Bloom gel strength is an important physical property of gelatin [9]. The bloom gel strength of the extracted gelatin was 8.1 and 0.03 (g) for samples G1 and G2, and close to 30.7 and 50.7 for the samples G3 and G4, respectively. In the literature, different values have been reported for gelatin extracted from the skin of other fish species: black tilapia (181 g), red tilapia (128 g) [10], cod (70 g), hake (100 g), megrim (340 g), sole (350 g) [11], and young Nile perch (81–222 g) and adult Nile perch (134–229 g) [12]. This variation depends on many factors, like different compositions of amino acids, the size of protein chains, and the concentration and molecular weight distribution of the obtained gelatin [11]. The analysis of macroelement composition (Na, Mg, K and Ca) revealed two different groups: samples G1 and G2, and samples G3 and G4. High levels of macroelements were found in G3 and G4 compared to the commercial samples G1 and G2. Regarding the microelements (Mn, Fe, Co, Ni, Zn, Cu, Se, and Sr), a higher quantity of Sr was observed for G3 (>60 mg/kg), whereas G4 presented a level of Zn 10 times higher than that of the other samples. Concerning the toxic elements (Cd, Hg, and Pb), we observed low concentrations, and consequently, no safety issues related to the samples were identified.

### 2.2. SDS-PAGE

**Figure 1:**
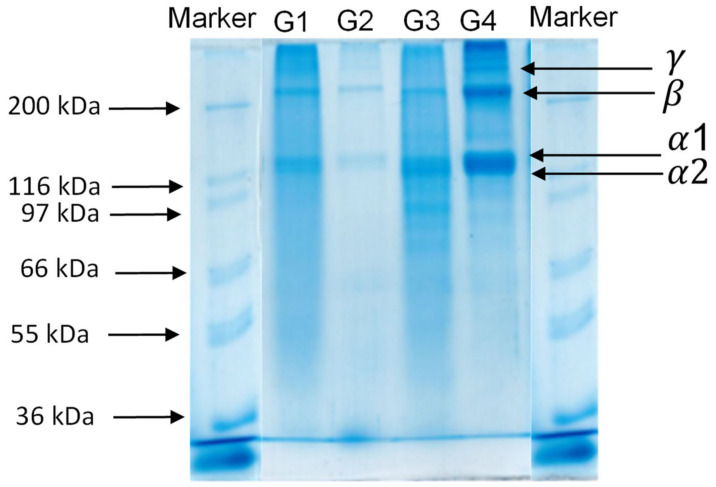
Protein profile for gelatin G1, G2, G3 and G4 with respective markers.

Polyacrylamide gel electrophoresis in the presence of SDS was employed to analyze the protein profile of the gelatin samples. The molecular weight distribution of samples G1, G2, G3 and G4 is compared and presented in Figure 1. As observed in our previous study [13], sample G1 presents an extensive hydrolysis of collagen. We can observe the α and β band for G1, G2 and G3 and only the *γ* band in G4. All samples showed electrophoresis patterns typical of type I collagen.

### 2.3. Amino Acid Composition

The amino acid compositions of samples G1, G2, G3 and G4, expressed as mg/g sample, are presented in Table 2. The properties of gelatin are influenced by the amino acid composition [14]. The most abundant amino acid present was glycine, followed by proline. In addition, a high content of alanine and glutamic acid was observed. All the samples present low contents of methionine, isoleucine, and tyrosine. The amino acid contents of samples G1, G2, G3 and G4 were 128, 199, 149 and 147 mg/g sample, respectively.

### 2.4. FTIR

FTIR peak locations are shown in Table 3, whereas the spectra are presented in Figure S1 (Supplementary Materials Figure S1). Generally, gelatin samples present five distinct regions for FTIR spectra: 3600–2300 (amide A), 3069–2940 (amide B), 1641–1633 (amide I), 1536–1516 (amide II) and 1270–1233 cm^−1^ (amide III). A free NH stretching vibration occurs in the range of 3400–3440 cm^−1^; however, when the NH group of a peptide is involved in a hydrogen bond, the position is shifted to around 3300 cm^−1^ [15]. Based on these results, it can be concluded that the NH groups of this gelatin were involved in hydrogen bonding, presumably with a carbonyl group of the peptide chain. The amide B band was split into two peaks for all samples due to asymmetric and symmetric C-H stretching. The amide I, II and III bands are responsible for the degree of molecular order found in collagen and involved in the formation of triple helical structure due to N-H bending and C-H stretching [16]. Amide I has frequencies from 1600 to 1700 cm^−1^, which are mainly associated with the stretching vibrations of the carbonyl group C=O. The frequency range of 1566 to 1636 cm^−1^ represents the β-sheet structure [17]. The wavenumber in the ranges of 1550–1540 cm^−1^ and 1525–1520 cm^−1^ correspond to the α-helical and β-sheet structures, respectively, caused by the amide II region. According to our results, peak wave numbers can be observed at 1520 for the G1 sample and 1522 cm^−1^ for the G2, G3 and G4 samples, respectively. According to Benjakul et al. [18], this band is typically more sensitive to hydration than to structural changes in protein. The amide III band was detected at 1233 cm^−1^ for G1 and G4 and 1244 and 1222 cm^−1^ for G2 and G3, respectively. Amide III represents the combination of the peaks for C-N stretching vibrations and N-H deformation from amide linkages and absorptions arising from the wagging vibrations of CH2 groups from the glycine backbone and proline side-chains [19].

### 2.5. Thermal Properties (DSC)

DSC analysis provides a sensitive means of understanding the heat-induced denaturation of gelatin samples [20]. Samples are equilibrated at 11% RH and characteristic transition temperatures for the samples G1, G2, G3 and G4 are presented in Table 4. DSC thermograms are presented in Figure S2 (Supplementary Materials). Tg was observed between 77 and 96 °C for all samples. Rahman et al. [21] observed Tg of extracted gelatin from Tuna skin at −24 °C, whereas Al-Saidi et al. [22], for shaari fish skin, found Tg at 15 °C. According to Al-Saidi et al. [22], the wide variation in Tg values may be due to the transformation of different types of gelatin using different extraction methods. Samples G1 and G3 present an unfolding temperature between ~143 and ~146 °C, whereas samples G2 and G4 present temperatures of ~156 and ~121 °C, respectively. The shift of ~20 °C between G3 and G4 is probably due to the pre-treatment in NaOH during the extraction process. Gelatin is a very complex molecule, and its thermal characteristics depend on many factors, including collagen type, tissue, animal species, and age [23]. The value of melting temperature varied between 157.3 °C for G4 and 189.2 °C for G2. According to Safandowska and Pietrucha [24], these results indicate that the breakage of peptide chains and subsequent destruction of materials takes place at various temperatures for the gelatin samples. The enthalpy of melting (ΔH (J/g)), determined from the area under the endothermic peak, was between 2.1 (G3 and G4) and 3.5 (G2), with a value of 2.7 for G1. The reduction in those peaks indicates that the proteins under consideration had partially lost their native form [15].

### 2.6. O/W Emulsions: Dh, ζ-Potential, and Physical Stability

**Figure 2:**
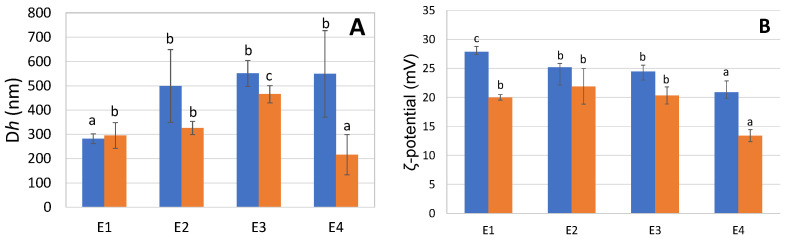

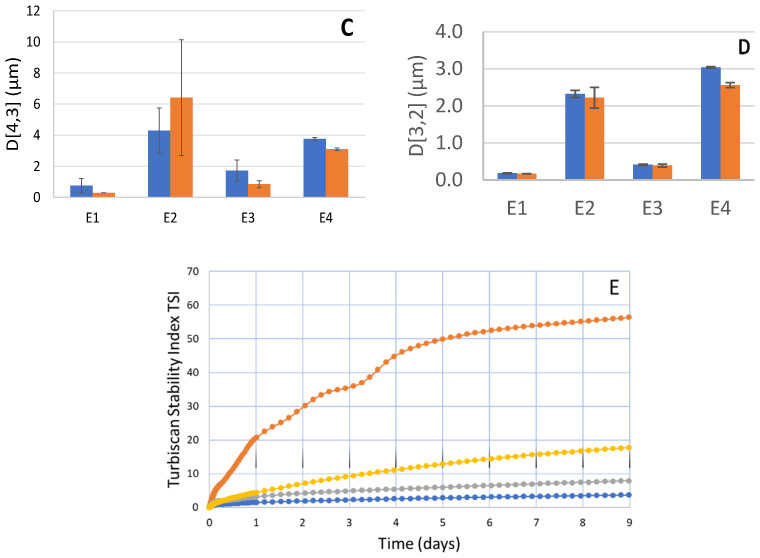
(**A**) Hydrodynamic diameter (Dh) and (**B**) ζ-potential for O/W emulsions, (**C**) surface-weighted mean, and (**D**) volume-weighted mean for E1, E2, E3 and E4 at day 1 (in blue) and day 9 (in orange). Letters a–c indicate significant differences between emulsions (*p* < 0.05). (**E**) Turbiscan stability index (TSI) as a function of time (days) for E1 (●), E2 (●) E3 (●) and E4 (●).

Figure 2A–C present the hydrodynamic diameter, the ζ-potential, and the physical stability of O/W emulsions E1, E2, E3 and E4. The droplet size of E1 changed from 282 to 296 nm over 9 days of storage (Figure 2A). Similar results were observed by Henriet et al. [7], where the authors observed an average size around 200 nm on day 0. E2, E3 and E4 present particle sizes ranging between 500 nm and 550 nm at day 1, with a significant tendency to decrease over 9 days except for E2, where there is no statistically significant change in the values. This is probably due to the different gelatin extraction protocols applied. The droplet sizes obtained here are smaller than those found in the study of Taherian et al. [25] at pH 3.4. Indeed, in a system comparable to the one used here (low fish oil content O/W emulsion stabilized with fish gelatin), they obtained a higher average particle size on day 1, with a value of 612.5 nm. However, in comparison with their O/W emulsion stabilized with whey protein isolate, it can be seen that these type of proteins exhibit a smaller droplet size at day 1 (338.9 nm) [25]. The charge of particles is related to the van der Waals and repulsion forces which could also affect the stability of emulsions [26]. ζ-potential results are illustrated in Figure 2B. First, we observe that the proteins provided positive charges, which was expected for emulsions prepared in buffer solution at pH 3, which is below the pH of gelatin proteins (pH ~6.7). In addition, all samples had ζ-potential values ranging between +20 and +28 mV, which is in agreement with the results reported by Henriet et al. [7]. Moreover, Taherian et al. [25] observed ζ-potential values of 42.7 ± 1.1 and 18.0 ± 0.4 mV for O/W emulsions stabilized with fish gelatin and whey protein isolate, respectively. ζ-potential values above +30 or lower than −30 mV are indicative of good emulsion stability [26]. E4 has a ζ-potential value of around 20 mV, with no statistically significant difference between days 1 and 9, which is significantly lower compared than the other emulsions (E1, E2 and E3), indicating the occurrence of less electrostatic repulsion between oil droplets in E4. E1–E3 were not significantly different at day 9, even though E1 had significantly higher ζ-potential at day 1 compared to E2 and E3. The differences between the samples can be attributed to the overall charge of the gelatins. Moreover, the presence of impurities such as free fatty acids from the fish oil could also have influenced the charge [27]. Figure 2C,D show the surface-weighted mean diameter (D [3,2]) and volume-weighted mean diameter (D [4,3]) of the emulsions. E1 and E3 possess much smaller droplets than E1 and E4. Droplet size distributions are shown in Figure S3, in the Supplementary Materials. All emulsions had a monomodal distribution on day 1. However, after 9 days of storage, E2 exhibited a bimodal distribution, indicating that coalescence had occurred by droplets merging and forming larger ones (Figure S3, Supplementary Materials). The physical stability, measured using the Turbiscan Tower, is presented in Figure 2E. It can be observed that emulsions E4 and E2 are less stable than E1 and E3. Based on Delta Back Scattering (ΔBS) (Figure S4, Supplementary Materials), which describes the backscattering of droplets over 9 days, different observations were made. For every sample, at the bottom of the cell, which is illustrated at the very left side of the graph, the ΔBS curves increase and move to the top of the cell, on the very right side of the graph. This phenomenon is called creaming, and can be explained by the lower density of fish oil compared to the water phase [7]. Creaming was improved when the emulsion contains smaller droplets. In the middle part of the cell, ΔBS decreases between 1000 μm and 3000 μm. This phenomenon can be attributed to coalescence or flocculation. It occurs due to the proteins lacking sufficient strength to cover the whole interfacial layer oil/water or to repulse other droplets. This phenomenon was observed for the emulsion stabilized with E3, but only after 9 days. All of the samples are monodispersed, but E2 is polydispersed for droplets above 2500 μm. This is probably due to the presence of foam during emulsification.

### 2.7. Oxidative Stability

#### 2.7.1. Peroxide Value

**Figure 3:**
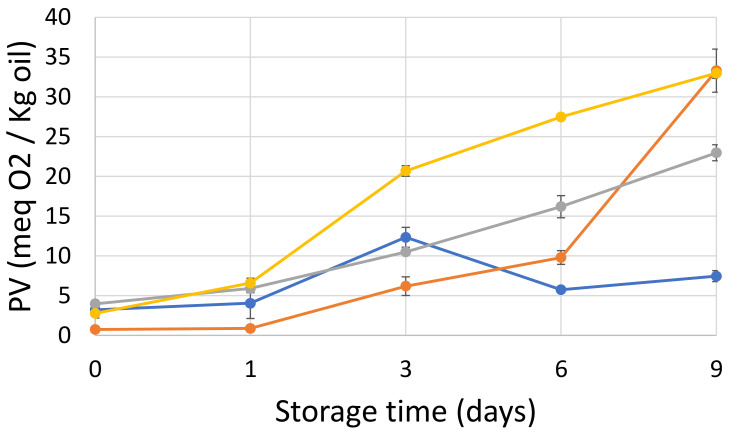
Peroxide value (meq O_2_/kg oil) as a function of time (day) for E1 (●), E2 (●) E3 (●) and E4 (●).

The peroxide value (PV) is a good indicator for the measurement of hydroperoxides, which are primary oxidation products. As can be seen in Figure 3, PV reached its highest level for the emulsions E2 and E4 (ca. 33 meq O_2_/kg oil), and then E3 (23.0 meq O_2_/kg oil) and E1 (7.5 meq O_2_/kg oil), at the end of the storage (day 9). At day 0, E4, E1 and E3 had a high PV after production (2.78, 3.24 and 4.00 meq O_2_/kg oil, respectively) which indicates that lipid oxidation had already occurred during homogenization. E1 exhibited a significant increase between days 1 and 3, and then a significant decrease after day 3, which could be due to the decomposition of hydroperoxides into secondary oxidation products. The PV of E2 and E3 significantly increased after day 1 and kept increasing until the end of storage. On the other hand, the PV of E4 already began increasing on day 0, and continued to increase until the end of the storage. Therefore, the lag phase was shorter for E4 than for the rest of the emulsions. These results are comparable with the results reported by Garcia-Moreno et al. [2], where the oxidative stability of 50 mg/g fish oil-in-water emulsions stabilized with hydrolyzed fish muscle proteins was investigated. The PV results ranging between 15 and 25 meq O_2_/kg oil on day 7 of storage were similar to those of our emulsions stabilized with non-commercial gelatin. When we compared gelatin as a stabilizer to other stabilizers (e.g., casein) in low-fat emulsions at pH 3, we found that the PV for casein-stabilized emulsions was much lower after 8 days of storage (~6 meq O_2_/kg oil) [28].

#### 2.7.2. Tocopherol Consumption

**Figure 4:**
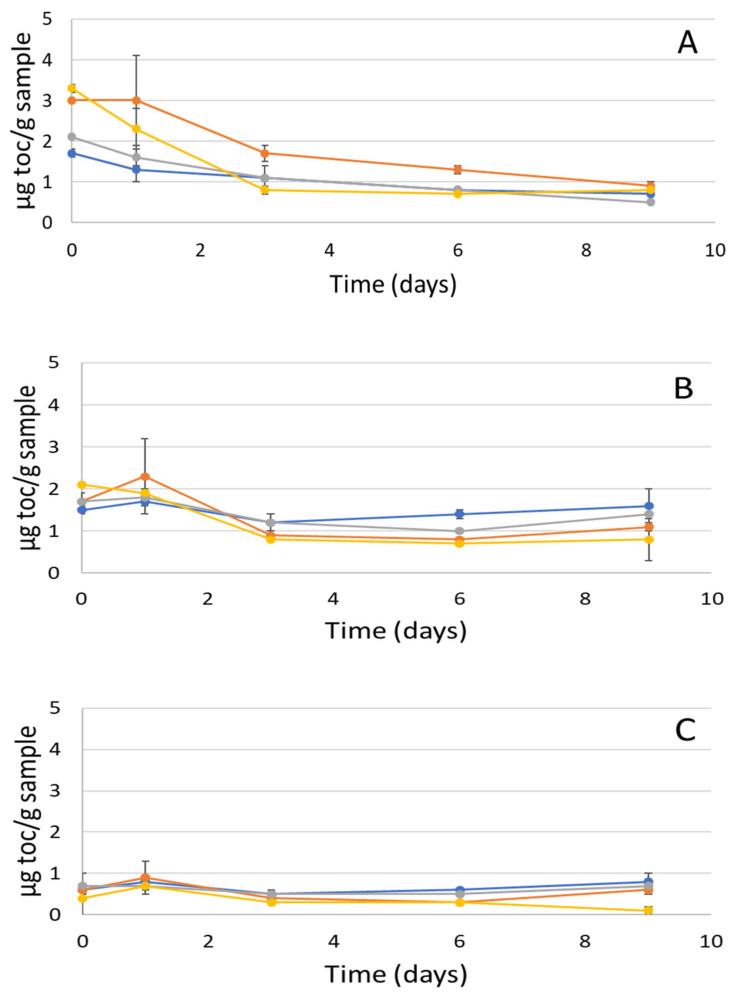
(**A**) Alpha-, (**B**) gamma- and (**C**) delta-tocopherol content (µm/g sample) as a function of time (day) for E1 (●), E2 (●) E3 (●) and E4 (●).

Tocopherols are natural antioxidants present in fish oil that are consumed as the level of oxidation increases. Thus, the decrease in the tocopherol content in the emulsions indicates an increase in oxidation. Therefore, changes in tocopherol content were monitored during the storage of the emulsions. Figure 4 presents the consumption of alpha-, gamma- and delta-tocopherols. Among the tocopherols, alpha-tocopherol exhibited the greatest decrease in concentration. Additionally, it has been reported that alpha tocopherol is important, as it accounts for about 90% of the tocopherols in animal tissues and has the highest biological influence [29]. After homogenization, E2 and E4 had significantly higher concentrations of alpha tocopherol (3 and 3.3 µg toc/g sample, respectively) than E1 and E3, as a result of the different levels of oxidation occurring during homogenization. The largest amount of alpha-tocopherol consumption was obtained for E4, followed by E2 and E3 (Figure 4A). These results are in line with the formation of hydroperoxide based on PV (Section 2.7.1). Similarly for gamma-tocopherol, the concentration decreased the most in E4 between days 0 and 9, followed by E2 and E3 (Figure 4B). Even though consumption of delta-tocopherol was minimal compared to the others, E4 exhibited the largest decrease in concentration (Figure 4C). Significant oxidation of E4, as reported in the previous section on the basis of PV results, was confirmed by the decrease in tocopherol results. The greatest decrease was observed from day 1 to day 3, followed by a further decrease until the tocopherols were almost depleted. A similar observation can be made for E2; the alpha-tocopherol content decreased the most when the PV increased the most between days 0 and 3 (Figure 4A). E1 and E3 exhibited a smaller reduction in tocopherols, which is in line with what was observed previously for PVs. These results showed that E1 and E3 were more oxidatively stable, since they did not require the consumption of these antioxidants in the fish oil. It is worth mentioning that alpha-tocopherol amounts decreased during storage, which is similar to the results reported by García-Moreno et al. [2] and Gelichi et al. [29]. It was noted that alpha- and gamma-tocopherol inhibit formation and decomposition of hydroperoxides [29]. Tocopherol levels in the present study were lower compared to the tocopherol amounts in 50 mg/g oil-in-water emulsions stabilized with common carp roe protein hydrolysate (CRPH) of [29] with 10 µg α-toc/g, samples, 5 µg *γ*-toc/g and 21 µg δ-toc/g after 7 days of storage, which was also due to having a larger amount of fish oil in the emulsion (50 mg/g).

#### 2.7.3. Secondary Volatile Oxidative Stability

**Figure 5:**
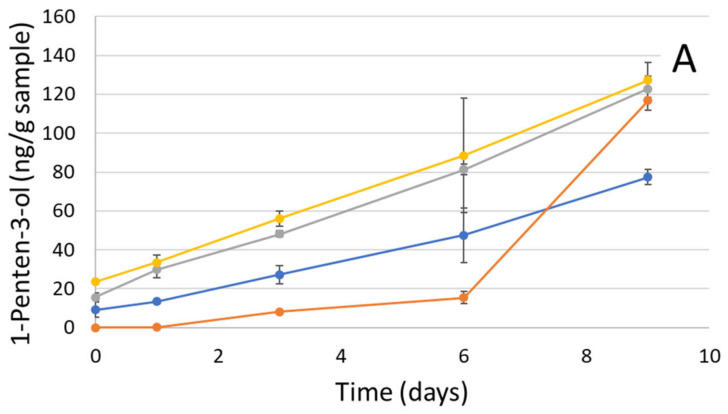

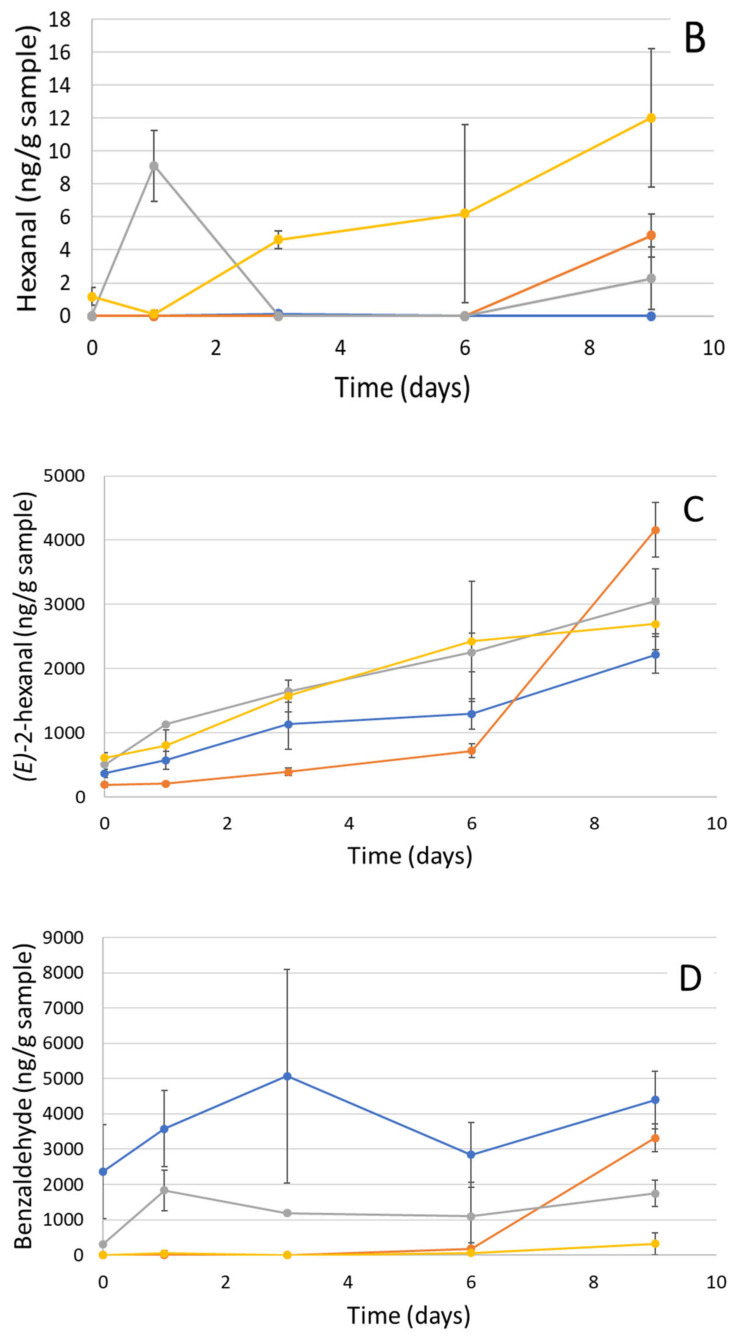
Development of the volatile compounds during 9 days of storage. (**A**) 1-penten-3-ol, (**B**) hexanal, (**C**) (E)-2-hexenal, and (**D**) benzaldehyde, for E1 (●), E2 (●) E3 (●) and E4 (●).

The formation of volatile compounds due to the decomposition of primary products is illustrated in Figure 5. Eleven volatile compounds were identified, detected in measurable amounts, and quantified during the storage of emulsions. Among them, 1-penten-3-ol, hexanal, (E)-2-hexenal, and benzaldehyde were chosen, as they were in high concentrations and exhibited trends representative of the rest of the volatile compounds. Briefly, some of the highest concentrations detected were as follows: benzaldehyde formed at concentrations of up to ~5000 ng/g sample in E1, followed by (E)-2-hexanal, at ~4000 ng/g sample in E2, and 1-penten-3-ol, at 130 ng/g sample in E4. Hexanal was detected at a concentration of 12 ng/g sample in E4. When these values were compared with the results reported by Haahr and Jacobsen [28], similar results were found for the emulsions stabilized with casein for 1-penten-3-ol after 8 days of storage (~127 ng/g sample), whereas much less was detected for (E)-2-hexanal (19.6 ng/g sample). Furthermore, Ghelichi et al. [29] presented similar concentrations for 1-penten-3-ol (<50 ng/g sample) and hexanal (<10 ng/g sample) in 50 mg/g fish oil-in-water emulsions stabilized with carp roe protein hydrolysate during a seven-day storage period. Generally, E2 and E4 had a higher increase in volatile concentration, such as 1-penten-3-ol, hexanal and (E)-2-hexenal, compared to E1 and E3. Our results are in agreement with those presented in the study of Haahr and Jacobsen [28], suggesting that the development of most volatiles exhibited a similar trend to that of the development of PV and the consumption of tocopherols. Moreover, E2 and E4 were more oxidized towards the end of storage. On the other hand, the formation of benzaldehyde was higher for E1 and E2 than for E3 and E4 on day 9. However, there were no significant differences among sampling days during 9 days of storage for E1, suggesting that the high levels of benzaldehyde were already present on day 0. Moreover, E2 had a low benzaldehyde concentration at the beginning, and the amount increased to almost the same level as E1 towards the end of the storage period. Overall, E2 is most stable at the beginning of the storage period, and a longer induction period was observed, meaning that it took longer (until day 6) for the secondary volatile oxidation products to form than for the other samples. This is presumably due to the compositional properties of the G2. When E1 and E3 are evaluated in comparison to E4on the basis of droplet size and zeta potential results, it can be observed that smaller droplets and higher zeta potential give better oxidative stability (Figure 2). In agreement with the studies of Ries et al. [30], Garcia-Moreno et al. [2] and Lethuaut et al. [31], emulsions with small droplets were more oxidatively stable. Indeed, an increase in the total droplet surface permitted more proteins for the protection of the oil droplets, thus enhancing the efficiency of the antioxidative effect of proteins. However, smaller droplets lead to a larger surface area and may also enhance oxidation. Nevertheless, other factors might also affect oxidative stability. Moreover, interaction with molecules from the gelatin samples may give rise to different volatile compounds. For instance, interaction with amphiphilic amino acids of the hydrolysates in the study by Ghelichi et al. [29] gave rise to 2-butanone. Additionally, another example by Ghelichi et al. [29] underlined microbial spoilage as being responsible for the formation of alcohols.

## 3. Discussion

The objective of this study was, in the first place, to compare the physico-chemical properties of four gelatins obtained using two different extraction protocols (G3 and G4) and two commercial proteins (G1 and G2). In the second part, we created the corresponding O/W emulsions E1, E2, E3 and E4, and investigated their physical and oxidative stability. Regarding their physico-chemical compositions, only sample G1 presented a high moisture content. This is probably due to the separation efficacy of the purification and drying methods. Otherwise, no significant differences were observed in terms of protein and ash content, or with respect to mineral composition. SDS-PAGE for gelatin extracted from saithe skin showed electrophoresis patterns typical of gelatin belonging to type I. The amino acid contents varied between 128 to 199 mg/g sample. This is probably due to differences in living environment, habitat, temperature and season [32]. The absorption bands of gelatin in the FTIR spectra were mainly situated in the amide band region (amide A, amide B, amide I, amide II and amide III), whereas DSC thermograms exhibited typical gelatin behavior. The results obtained in Turbiscan showed that emulsions E1 and E3 were physically more stable than E2 and E4. Indeed, they had smaller particles and exhibited a smaller decrease in ζ-potential between day 1 and 9 than the others. With respect to oxidative stability, even though the development of secondary volatile oxidation compounds took place more slowly in the case of E2 until day 6 than in the other emulsions, both E2 and E4 demonstrated the highest formation of oxidation products at the end of the 9-day storage period. E1, followed by E3, exhibited slower formation of hydroperoxides and some volatile compounds, indicating promising antioxidant properties. Thus, it can be concluded that emulsions E2 and E4 were physically and oxidatively less stable than emulsions E1 and E3. These results provide new perspectives on the possible application of the G3 as a new emulsifying or microencapsulating agent in food and cosmetic products. In addition, further investigations using a pendant drop in static and dynamic mode, as well as with a double-wall ring rheometer will be necessary to obtain more information regarding properties at the O/W interface.

## 4. Materials and Methods

### 4.1. Raw Material

Sample G1 consists of gelatin from cold water fish skin, obtained from Sigma (Sigma, St. Louis, MO, USA). Sample G2 consists of commercial gelatin Byco M, kindly donated by Croda (Snait Gole, East Yorkshire, UK). Samples G3 and G4 were obtained from saithe (Pollachius virens) skin, as described in Table 5. Fish oil was supplied by Vesterålens A/S (Sortland, Norway) and stored at −40 °C. The peroxide value of the fish oil was 0.18 ± 0.00 meq O_2_/kg oil. Alpha-, gamma- and delta-tocopherol contents were respectively, 257 ± 2, 144 ± 1.6 and 62 ± 0.7 µg toc/g oil.

### 4.2. Chemical Composition

Moisture and ash contents were determined according to AOAC standard methods 930.15 and 942.05, respectively [33]. Total nitrogen content was determined using the Dumas method [34]. A conversion factor of 5.55 was used for the conversion of nitrogen to protein content.

### 4.3. Determination of Gel Strengths (Bloom Strength)

Gel strength of the samples was determined using the method described by Wainewright [35], Bath et al. [36] and Casanova et al. [13].

### 4.4. Mineral Composition

The mineral composition of the samples was determined following the analytical principles in the European standards EN13805 and EN15763. Briefly, ~0.3 g subsamples were first digested with 5 mL of concentrated nitric acid (SPS Science, Paris, France) in a microwave oven (Multiwave 3000, Anton Paar, Graz, Austria). Then, the digests were diluted with ultrapure water (Millipore, Milford, MA, USA). Subsequently the mineral composition was determined using inductively coupled plasma mass spectrometry (iCAPq ICPMS, Thermo Fisher Scientific, Waltham, MA, USA) according to Casanova et al. [13].

### 4.5. Electrophoretic Study (SDS-PAGE)

Polyacrylamide gel electrophoresis was used for the determination of sample protein profiles using a Mighty Small (Hoefer) slab cell as described by Laemmli [37], using 12% acrylamide (C = 26 mg/g) slab gels (1.5 mm thick), as reported by Casanova et al. [15]. Electrophoresis was carried out at 100 V for 15 min, followed by 150 V for 1 h (max. 40 mA per gel). Afterwards, the gel was stained using colloidal Coomassie Brilliant Blue as described by Rabilloud and Charmont [38]. Mark12^TM^ from Novex was used as molecular weight markers.

### 4.6. Amino Acid Composition

The amino acid profile was measured according to Casanova et al. [13]. Briefly, 10 mg sample/mL HCl (6 M) was hydrolyzed in an oven for 18 h at 110 °C. Then, the samples were cooled to room temperature and used both without dilution and after a 3-fold (1 + 2) dilution with 6 M HCl during the remaining procedure in order to quantify both high- and low-abundance amino acids. Then, 100 μL was diluted with 1.5 mL 1 M NaCO3 and filtered through a 0.2 μm syringe filter (Q-max PTFE, Ø13 mm, Frisenette ApS, Knebel, Denmark) before derivatization using the EZ: Faast™ Amino Acid Analysis kit from Phenomenex^®^ (Torrance, CA, USA). The samples (50 μL) were then analyzed by LC-(APCI)-MS (Agilent 1100, Agilent Technology, Santa Clara, CA, USA) according to the procedure described by Farvin et al. [39].

### 4.7. Fourier Transform Infrared (FTIR) Spectroscopy

Equilibrated gelatin samples (11% RH) were analyzed by FTIR using a PerkinElmer Spectrum 100 FT-IR spectrometer (Waltham, MA, USA) between 650 and 4000 cm^−1^ at a resolution of 4 cm^−1^. Automatic signals were recorded in 4 scans. Measurements were performed in triplicate at room temperature (20 ± 1 °C).

### 4.8. Differential Scanning Calorimetry (DSC)

The gelatin samples (11% RH) were measured with respect to their unfolding, glass transition, and solid melting temperatures by means of differential scanning calorimetry DSC 250 (TA Instruments, New Castle, DE, USA) equipped with a Refrigerated Cooling System 90, as described by Casanova et al. [13]. Thermograms were analyzed using Trios software. Measurements were performed in triplicate.

### 4.9. Preparation and Physical Stability of O/W Emulsions

O/W emulsions, namely E1, E2, E3 and E4, were prepared with the gelatins G1, G2, G3 and G4, respectively, using the method described by Henriet et al. [7], with some modifications. Briefly, 4 wt.% protein was dissolved in citric acid buffer at pH 3 overnight (20 °C) and 2 wt.% fish oil-in-water emulsions were produced using Ultra-Turrax (DI 25 Basic, IKA, Staufen, Germany), followed by ultrasonication (SFX550 Model, Branson Ultrasonics Corp., Danbury, CT, USA). The protein solution was homogenized for 10 min (2.5 min × 4) at 8000 rpm, with oil being added slowly, followed by additional blending for 10 min (2.5 min × 4) at 20,500 rpm. To avoid an increase in temperature, the beaker was placed in an ice-water bath. Ultrasonication was applied for 20 min at 550 Hz in pulsed mode (10 s ON and 10 s OFF) at 100% amplitude while being kept in the ice-water bath. The storage experiment was carried out by storing the emulsions in brown bottles in darkness at room temperature for 9 days. Physical stability was measured at 20 °C using a Turbiscan tower (Formulaction, Toulouse, France) over 9 days. A volume of 20 mL of fresh emulsion of each sample was transferred into three different cells by using syringe to avoid any significant spot on the glass surface. The vial was placed into the Turbiscan tower, and the physical stability of the emulsion was checked by the scanning program over 9 days as follows: 1 scan every 5 min during the first 6 h; 1 scan every 10 min between 6 and 12 h; 1 scan every 1 h between 12 and 24 h; 1 scan every day between 1 and 9 days.

#### 4.9.1. Hydrodynamic Diameter by Dynamic Light Scattering (DLS)

Hydrodynamic diameter (Dh) was determined by DLS on a Zetasizer Nano-S (Malvern Instrument, Worcestershire, UK). Measurements were performed at a scattering angle of 173° and a wavelength of 632.8 nm. Fresh emulsions were first diluted 500 times using deionized water. The Dh of the particles was calculated using the Stokes–Einstein equation on the basis of the diffusion coefficient (Dt) extracted from the fit of the correlation curve using the cumulant method.

#### 4.9.2. ζ-Potential Measurements

ζ-potential (ζ) was determined using a Zetasizer Nano-S (Malvern Instrument, Worcestershire, UK) with capillary cells on days 1 and 9 of the storage. Emulsions were diluted 500 times in distilled water. The analyses were performed by applying a voltage of 50 mV. ζ-potential (ζ) was calculated from the electrophoretic mobility (μ) using Henry’s equation.

#### 4.9.3. Droplet Size Distribution

The droplet size of the emulsions was determined by laser diffraction using a Mastersizer 2000 (Malvern Instruments, Ltd., Worcestershire, UK) on days 1 and 9. Emulsions were diluted in recirculating water set at 3000 rpm until an obscuration of approximately 12–15% was reached. For the particle and the dispersant, the refractive indices of sunflower oil (1.469) and water (1.330) were used, respectively. The results were calculated as the surface-weighted (D [3,2]) and volume-weighted (D [4,3]) mean diameters.

### 4.10. Oxidative Stability

Lipid extraction, peroxide value determination, consumption of tocopherols and formation of secondary volatile oxidation products were analyzed on the sampling days 0, 1, 3, 6, and 9.

#### 4.10.1. Peroxide Value

Prior to peroxide value determination, fish oil was extracted from the emulsions according to the method of Bligh and Dyer using a reduced amount of the chloroform/methanol (1:1 *w*/*w*) solvent [40]. Two lipid extractions were performed from each emulsion and the peroxide value was determined on lipid extracts using the colorimetric ferric-thiocyanate method at 500 nm, as described by Shantha and Decker [41]. Measurements were performed in duplicate.

#### 4.10.2. Tocopherol Consumption

The tocopherol content was determined using about 2 g of the chloroform extract obtained from the Bligh and Dyer extraction, which was then evaporated under nitrogen and dissolved in 1 mL n-heptane. Samples were analyzed by HPLC (Agilent 1100 Series, Waldbronn, Germany; Column: Waters Spherisorb 3 μm Silica; 4.6 × 150 mm, Waltham, MA, USA). Analysis was performed according to the official AOCS method [41]. Measurements were performed in duplicate and quantified by authentic standards. Results are expressed in µg tocopherol per g of oil.

#### 4.10.3. Secondary Volatile Oxidation Products Using Dynamic Headspace GC-MS

Approximately 4 g of emulsion and 30 mg internal standard (4-methyl-1-pentanol, 30 μg/g water) were weighed in a 100 mL purge bottle. Distilled water (5 mL) and antifoam (1 mL) (Synperonic 800 μL/L water) were added into the purge bottle and heated in a water bath at 45 °C while purging with nitrogen (flow 150 mL/min) for 30 min. Tenax GR tubes were used for trapping the volatile compounds, desorbed again by heat (200 °C) in an Automatic Thermal Desorber (ATD-400, Perkin Elmer, Norwalk, CN, USA), cryofocused on a cold trap (−30 °C), released again (220 °C), and led to the gas chromatograph (HP 5890IIA, Hewlett Packard, Palo Alto, CA, USA; Column: DB-1701, 30 m × 0.25 mm x 1.0 μm; J&W Scientific, Santa Clara, CA, USA). The oven program had an initial temperature of 45 °C for 5 min, which was increased by 1.5 °C/min until 55 °C, by 2.5 °C/min until 90 °C, and by 12.0 °C/min until 220 °C, at which point the temperature was maintained for 4 min. The individual volatile compounds were analyzed by mass spectrometry (HP 5972 mass-selective detector, Agilent Technologies, Santa Clara, CA, USA; electron ionization mode, 70 eV; mass-to-charge ratio scan between 30 and 250), further identified by MS-library (Wiley 138 K, John Wiley and Sons, Hoboken, NJ, USA, Hewlett-Packard, Palo Alto, CA, USA), and quantified on the baiss of calibration curves. The external standards selected were 2-ethylfuran, 1-penten-3-ol, pentanal, (E)-2-heptenal, hexanal, (E)-2-hexenal, heptanal, (E,E)-2,4-heptadienal, nonanal, (E,E)-2,6-nonadienal. The standard solutions were injected directly into the emulsion that was produced, similarly to emulsion E1, and the calibration curves were obtained for quantification. Measurements were performed in triplicate for each sample.

### 4.11. Statistical Analysis

Statgraphics 18 (Statistical Graphics Corp., Rockville, MD, USA) was used for the statistical analysis. Mean values and standard deviations were used for the data analysis and multiple samples were compared in order to identify the significant differences between samples on certain sampling days and between sampling days for each sample during storage using Tukey as a post hoc test at *p* < 0.05 significance level.

## Figures and Tables

**Table 1:** Physico-chemical properties, gel strength and mineral composition of commercial fish gelatin G1 and G2 compared to extracted fish skin gelatin G3 and G4.

		G1	G2	G3	G4
Moisture (%)		11.73 ± 0.08	6 ± 0.09	5.08 ± 0.10	7.18 ± 0.06
Ash (%)		0.18 ± 0.03	0.09 ± 0.08	3.39 ± 0.11	2.05 ± 0.07
Protein (%)		95.36 ± 0.23	96.05 ± 0.81	95.01 ± 0	95.75 ± 1.05
Gel strength (g)		8.1 ± 2.9	0.03 ± 0	30.7 ± 11.70	50.7 ± 2.70
Macroelements (g/kg dry matter)	Na	0.08	1.32	1.90	9.33
	Mg	0.01	0.01	0.93	0.03
	K	0.16	0.07	2.75	0.72
	Ca	0.51	0.08	6.56	0.59
Microelements (mg/kg dry matter)	Cr	0.23	0.06	0.25	4.03
	Mn	0.01	0.031	0.18	0.59
	Fe	76.40	1.10	2.93	28.46
	Co	<0.01	<0.01	0.01	0.04
	Ni	0.06	0.03	0.12	1.89
	Zn	0.36	0.61	0.75	6.22
	Cu	0.45	0.28	0.19	1.65
	Se	4.57	0.19	0.72	0.66
	Sr	2.16	0.22	62.05	1.19
Toxic elements (mg/kg dry matter)	Cd	0.33	<0.01	<0.01	<0.01
	Pb	<0.05	0.01	<0.05	<0.04
	Hg	<0.01	<0.01	<0.01	<0.01

**Table 2:** Amino acid composition for the sample G1, G2, G3 and G4 expressed as mg/g sample.

	G1	G2	G3	G4
Phenylalanine	32.76 ± 4.08	47.18 ± 12.26	29.63 ± 13.33	25.50 ± 4.71
Leucine	15.53 ± 1.08	21.72 ± 0.64	15.80 ± 2.16	15.63 ± 1.42
Isoleucine	10.10 ± 0.49	11.72 ± 0.28	10.63 ± 1.33	10.27 ± 0.84
Methionine	12.44 ± 0.90	8.00 ± 0.30	12.51 ± 2.80	11.29 ± 1.29
Proline	83.52 ± 12.07	122.36 ± 11.12	101.19 ± 10.90	100.67 ± 6.58
Tyrosine	5.93 ± 0.07	3.57 ± 0.44	6.06 ± 0.92	5.83 ± 0.30
Valine	19.37 ± 0.33	22.03 ± 1.66	21.57 ± 2.38	21.12 ± 1.38
Alanine	71.96 ± 4.15	98.07 ± 4.20	73.60 ± 9.23	74.95 ± 2.03
Threonine	24.78 ± 0.90	23.96 ± 2.34	25.28 ± 3.06	24.85 ± 2.21
Glycine	207.40 ± 12.20	247.19 ± 8.84	216.84 ± 27.21	219.60 ± 7.98
Serine	49.26 ± 1.40	31.80 ± 1.85	52.70 ± 6.23	52.78 ± 3.80
Arginine	60.95 ± 0.34	79.75 ± 1.84	60.48 ± 8.49	63.57 ± 3.73
Histidine	8.40 ± 0.57	4.20 ± 0.86	8.07 ± 0.56	7.86 ± 1.05
Lysine	21.72 ± 2.65	23.84 ± 1.61	20.77 ± 2.94	21.04 ± 1.87
Glutamic acid	81.38 ± 2.64	90.30 ± 5.05	83.28 ± 10.30	86.60 ± 2.94
C-C	0.13 ± 0.09	0.05 ± 0.01	0.12 ± 0.02	0.10 ± 0.03
Aspartic acid	51.29 ± 1.60	50.12 ± 2.50	51.75 ± 5.68	53.60 ± 2.24
Hydroxyproline	45.06 ± 3.37	77.60 ± 3.26	48.64 ± 1.45	47.75 ± 1.60
TOTAL	8904.02 ± 49.94	936.60 ± 59.11	838.97 ± 109.04	843.05 ± 46.06

**Table 3:** FTIR peak locations for samples G1, G2, G3 and G4.

Region	Peak Wavenumber (cm^−1^)			
	G1	G2	G3	G4
Amide A	3277	3244	3277	3277
Amide B	3069	2966	3055	3066
	2944	2900	2955	2933
Amide I	1633	1566	1622	1636
Amide II	1520	1522	1522	1522
Amide III	1233	1244	1222	1233

**Table 4:** Temperature of glass transition (a), unfolding (b), and melting of solids (c) (°C), and the enthalpy of melting ∆H (J/g) for the samples G1, G2, G3 and G4.

Sample	a (°C)	b (°C)	c (°C)	∆H (J/g)
G1	77.1	146.3	168.1	2.7
G2	95.5	156.1	189.2	3.5
G3	95.8	143.6	161.2	2.1
G4	89.3	121.2	157.3	2.1

**Table 5:** Extraction protocols for the commercial samples G1, G2 and extracted fish skin gelatin G3 and G4.

Sample	Skin Washing	Pre-Treatment	Washing	Step 1	Step 2	Step 3	Step 4
G1	Commercial sample						
G2	Commercial sample						
G3	Water	neutrase in water for 10 min at 40 °C	Water	pH to 3.5 with citric acid (dry form) Incubated for 30 min	heated to 80 °C during 15 min and incubated for 5 min	centrifuged for 5 min at 2100× *g*	adjusted at pH 6.5 with NaOH
G4	Water	0.2 N NaOH for 60 min at 5 °C	Water	pH to 3.5 with citric acid (dry form) Incubated for 30 min	heated to 80 °C during 15 min and incubated for 5 min	centrifuged for 5 min at 2100× *g*	adjusted at pH 6.5 with NaOH

## Data Availability

Not applicable.

## Supplementary Materials

The following supporting information can be downloaded at: https://www.mdpi.com/article/10.3390/md20120739/s1, Figure S1: FTIR spectra of sample G1, G2, G3 and G4; Figure S2: DSC thermograms of sample G1, G2, G3 and G4. The letters a, b and c refer to the glass transition (Tg), unfolding and solids-melting temperature, respectively; Figure S3: Droplet size distribution data for the emulsions E1, E2, E3 and E4. The first graph shows the distributions on day 1 and the second graph shows the distributions on day 9.; Figure S4: Turbiscan ΔBS results for the samples E1, E2, E3 and E4 during 9 days of storage. Curve colors become lighter as the storage time is longer.
