# Supplementary material for: Gelatin from Saithe (Pollachius virens) Skin: Biochemical Characterization and Oxidative Stability in O/W Emulsions
Source: Mar Drugs. 2022 Nov 25;20(12):739. doi: 10.3390/md20120739 (PMC9785016; doi:10.3390/md20120739)
Supplement: Supplementary file 1 [file marinedrugs-20-00739-s001.zip › marinedrugs-1885796-supplementary.pdf]

Supplementary material

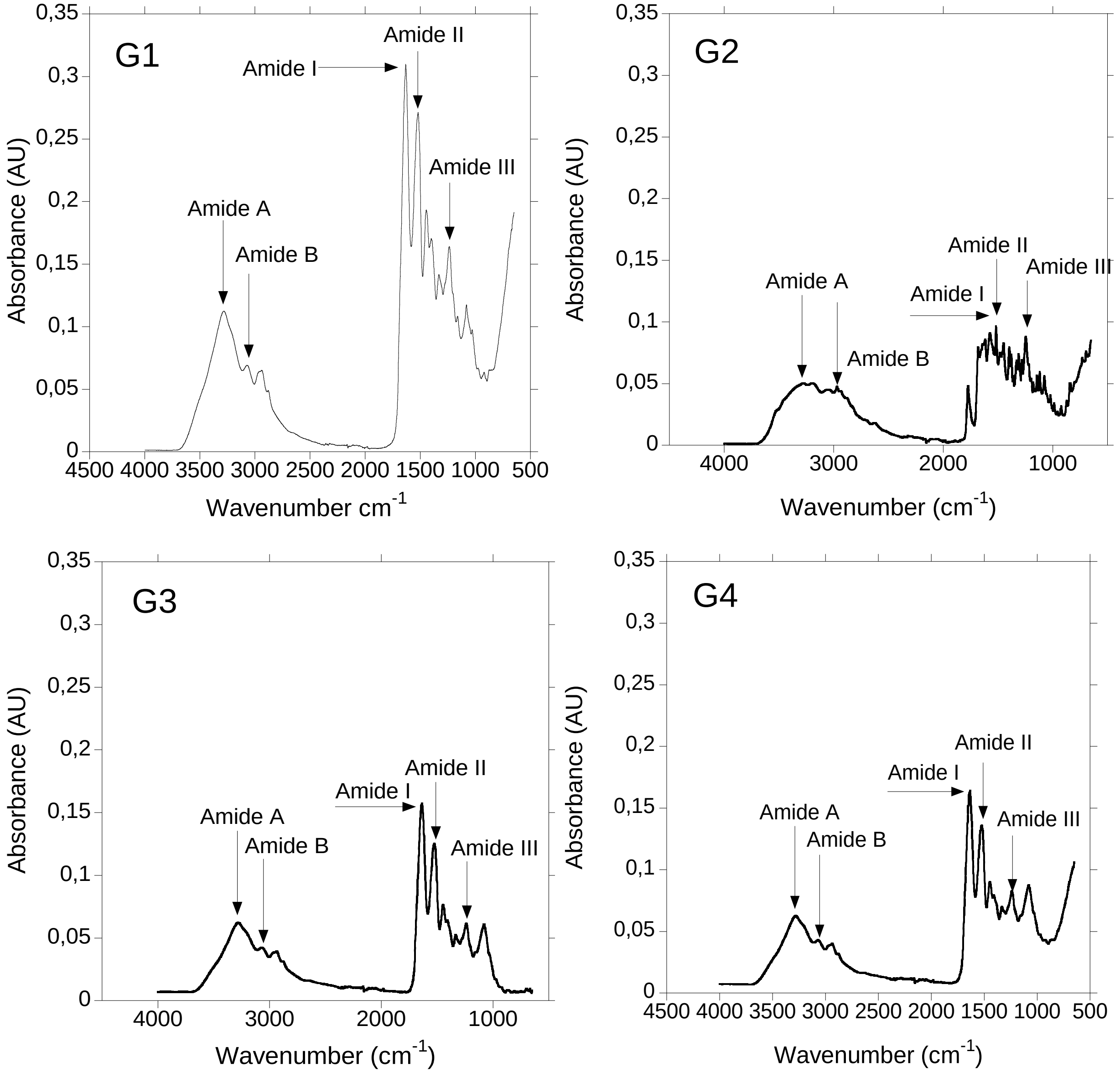

Figure S1. FTIR spectra of sample G1, G2, G3 and G4.

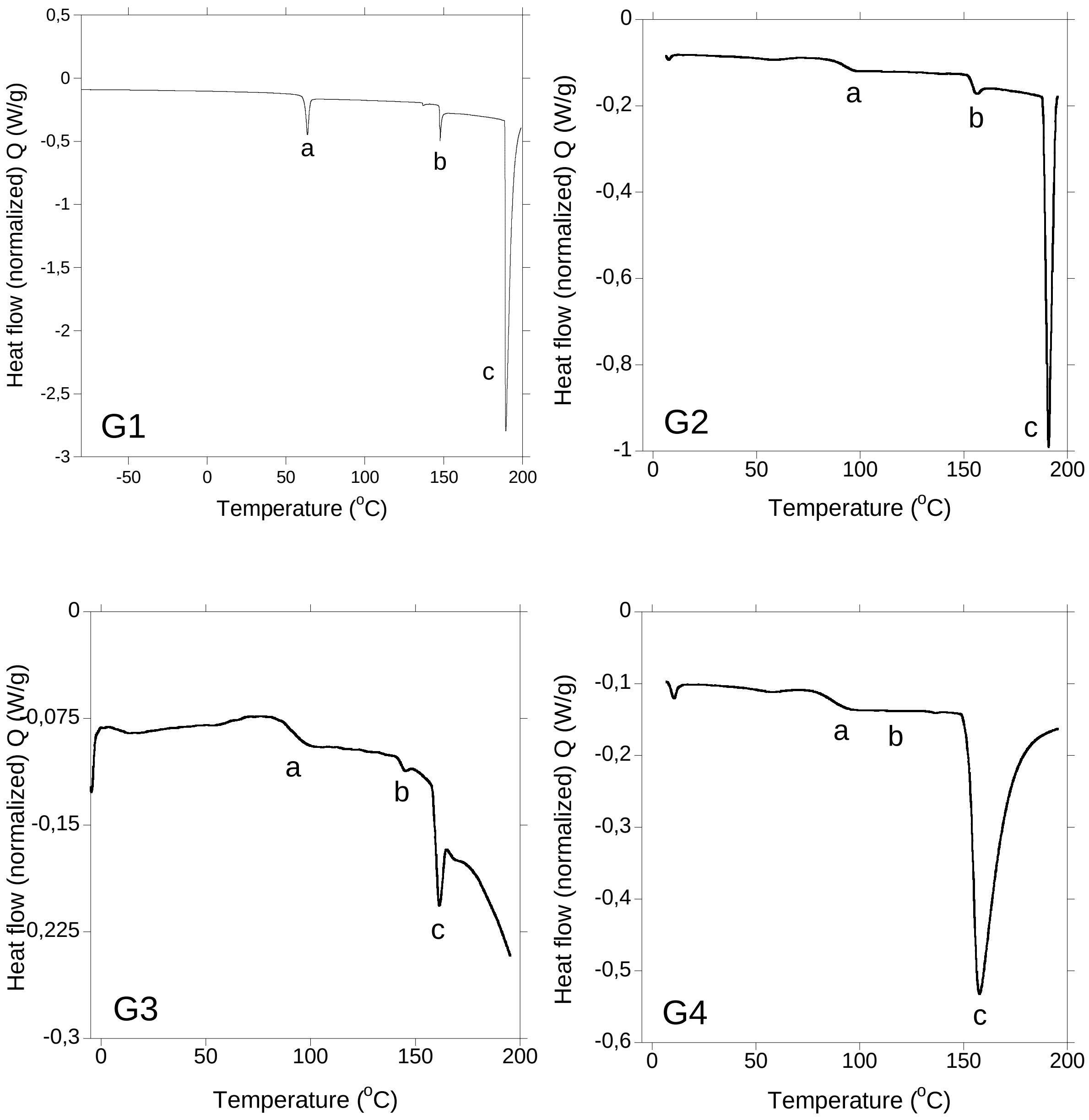

Figure S2. DSC thermograms of sample G1, G2, G3 and G4. The letters a, b and c refer to the glass transition (Tg), unfolding and solids-melting temperature, respectively.

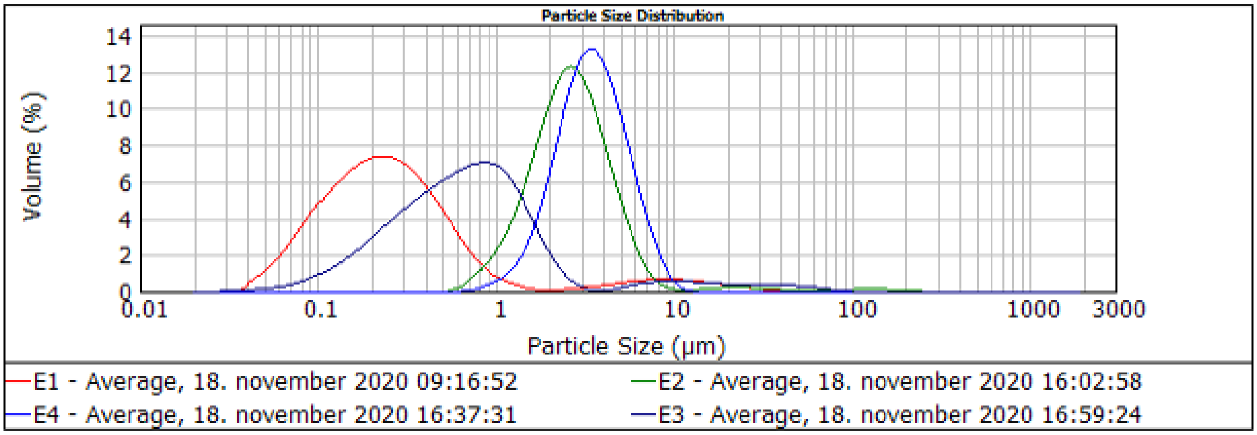

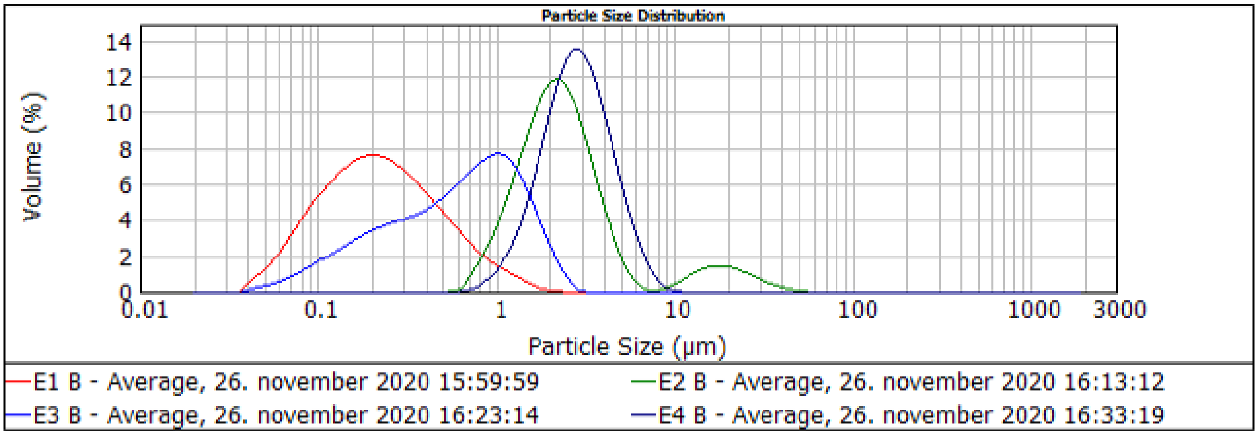

Figure S3. Droplet size distribution data for the emulsions E1, E2, E3 and E4.The first graph shows the distributions on day 1 and the second graph shows the distributions on day 9.

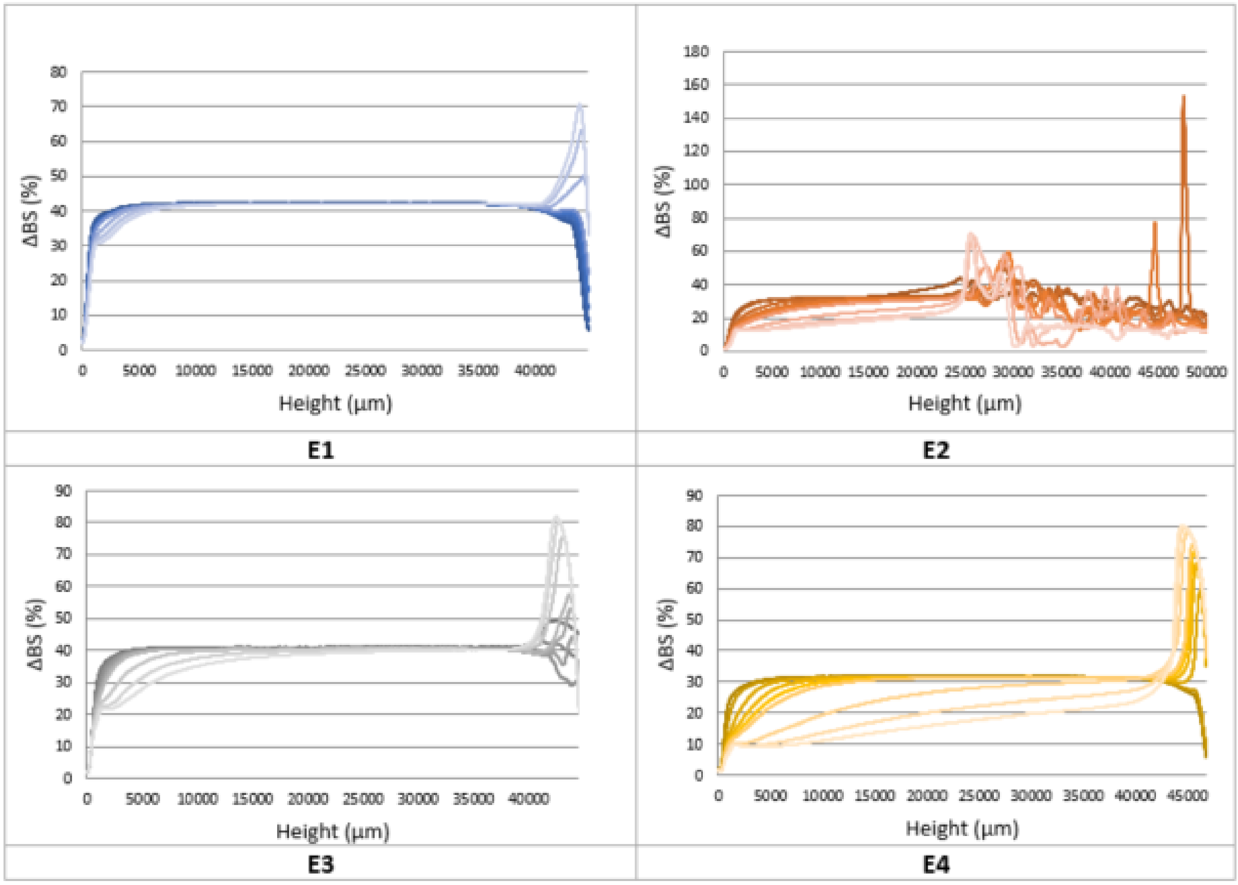

Figure S4. Turbiscan ΔBS results for the samples E1, E2, E3 and E4 during 9 days of storage. Curve colors become lighter as the storage time is longer.
